# Bone Marrow Iron Stores Are Not Associated with Increased Risk for Invasive Fungal Infections in Patients with Newly Diagnosed Acute Leukemia or Myelodysplastic Syndrome in Transformation: Is There a Relationship?

**DOI:** 10.3390/jof9070748

**Published:** 2023-07-14

**Authors:** Eirini A. Apostolidi, Maria N. Gamaletsou, Maria Arapaki, John V. Asimakopoulos, Panagiotis Diamantopoulos, Sofia Zafeiratou, Diamantis Kofteridis, Maria Pagoni, Maria Kotsopoulou, Michael Voulgarelis, Nikolaos V. Sipsas

**Affiliations:** 1 Pathophysiology Department, General Hospital of Athens Laiko, Medical School, National and Kapodistrian University of Athens, 11527 Athens, Greece; e-apostolidi@med.uoa.gr (E.A.A.);; 2 Hematology Department, General Hospital of Athens Laiko, 11527 Athens, Greece; 3 First Department of Internal Medicine, General Hospital of Athens Laiko, Medical School, National and Kapodistrian University of Athens, 11527 Athens, Greece; 4 Department of Hygiene, Epidemiology and Medical Statistics, Medical School, National and Kapodistrian University of Athens, 11527 Athens, Greece; 5 Medicine Department, Medical School, University of Crete, 71003 Heraklion, Greece; 6 Hematology Department, General Hospital of Athens Evangelismos, 10676 Athens, Greece; 7 Hematology Department, Metaxa Hospital, 18537 Piraeus, Greece

**Keywords:** invasive fungal infections, mycoses, acute leukemia, myelodysplastic syndrome, iron, iron overload, ferritin, aspergillosis, invasive candidiasis, mucormycosis

## Abstract

Iron plays an important role in the pathogenesis of infections, including invasive fungal infections (IFIs). Studies suggested that iron overload might represent an additional risk factor for IFIs among patients with hematological malignancies. We conducted a prospective, multi-center study amongst adult patients with newly diagnosed acute myeloid leukemia (AML) or myelodysplastic syndrome (MDS) in transformation to determine whether baseline iron overload as measured using the bone marrow iron store (BMIS) score is an independent risk factor for the development of IFIs. We also measured baseline serum iron and ferritin levels. A total of 98 patients were enrolled (76 with AML) and were followed for 12 months. Twenty-two patients developed IFI during the follow-up period (invasive aspergillosis *n* = 16, candidemia *n* = 5, mucormycosis *n* = 1). A baseline BMIS score ≥ 3 indicated that iron overload was relatively common (38/98 patients, 38%), and its frequency was comparable between patients with no IFIs (31/76, 40.7%) and in those with IFIs (8/22, 36.4%). Univariate analysis showed that only the presence of AML was associated with increased risk for IFIs [OR (95% CI) 7.40 (1.05–325.42)]. Both univariate and multivariate analyses showed that an increased BMIS score (≥3) at baseline was not an independent risk factor for IFIs. Similarly, there was no difference in serum iron and ferritin between the two groups that had similar demographic characteristics. Indices of iron overload were not independent risk factors for IFIs in our cohort of Greek patients with newly diagnosed AML/MDS in transformation.

## 1. Introduction

Despite progress, invasive fungal infections (IFIs) remained a feared complication of hematologic malignancy treatment [1]. Heavy immunosuppression, resulting from the hematologic malignancy itself or the available treatment modalities, such as intense chemotherapy, biologic agents, radiation therapy, and hematopoietic stem cell transplantation (HSCT), is the driving force for the susceptibility to IFIs. The role of the impaired innate immunity (a reduced number and function of neutrophils and macrophages) and the impaired T cell immunity is crucial, which is of paramount importance for the immune response to intracellular pathogens.

The incidence of IFIs is highest in patients with acute myeloid leukemia (AML) or myelodysplastic syndrome (MDS) in transformation because these patients are routinely treated with intensive chemotherapy resulting in protracting neutropenia, which is a well-established risk factor for IFIs [2]. Other risk factors include treatment with immunosuppressive drugs, corticosteroid use, co-infection with cytomegalovirus, graft-versus-host disease among recipients of HSCT, and active malignancy. Researchers have shown that the risk of IFIs is higher among non-responders to chemotherapy, relapsed patients, or patients with refractory disease [3]. Newer treatment modalities, such as biologic agents, are emerging as additional risk factors for IFIs [4,5,6]. IFIs do not develop in all high-risk patients, suggesting that other biological factors may contribute to the development of these dreadful infections [7,8,9]. Discovery of novel risk factors is important for designing prophylactic and early treatment strategies.

Iron plays an important role in the pathogenesis of infections, as it boosts the ability of pathogens to multiply in host cells, tissues, and organs [10]. Moreover, iron interferes with the immune response to infections [11]. Experimental data show that iron impairs in vitro the phagocytic, chemotactic, and bactericidal capacity of effector immune cells, such as neutrophils and monocytes, affects the generation of cytotoxic T-cells, markedly reduces the proliferative capacity of helper T-cells, and inhibits the activity of natural killer cells and macrophages [10,12,13].

Regarding mycoses, iron is also essential for the growth and virulence of a variety of fungal pathogens [14]. Like bacteria, fungi need iron for their survival, as this micronutrient is of paramount importance for biological processes such as DNA replication, transcription, metabolism, and energy generation, which facilitate the propagation of fungal infection [15,16]. Fungi developed several sophisticated mechanisms to acquire iron from their mammalian hosts, such as reduction of ferric to ferrous iron, siderophore production, iron acquisition from hemoglobin, use of xenosiderophores, and others [14,16]. In vivo studies in animal models have shown that iron plays an important role in the pathogenesis of invasive aspergillosis (IA), mucormycosis, and possibly invasive candidiasis [17,18]. On the contrary, iron starvation has been shown to induce apoptosis in *Mucorales*, in vitro and in experimental models [19,20].

Because patients with acute myeloid leukemia or transfusion-dependent myelodysplastic syndrome in transformation at a high risk of IFIs [21] routinely receive multiple units of red blood cells, transfusion-induced iron overload could represent an unrecognized risk factor for IFIs. Clinical studies indicate that iron overload might be a predisposing factor for IFIs in this high-risk population, but these studies are either small case series, retrospective studies, or use serum parameters to define iron overload, such as serum iron and ferritin levels, which are affected by inflammation [7,22,23]. We, therefore, conducted a prospective, non-interventional, multi-center study amongst patients with newly diagnosed acute myeloid leukemia or MDS in transformation to determine whether baseline iron overload, as measured using the bone marrow iron store (BMIS) score, before initiation of chemotherapy or other treatment, is an independent risk factor for the development of IFIs.

## 2. Materials and Methods

All consecutive patients with newly diagnosed AML or with transfusion-dependent MDS in transformation, before initiation of chemotherapy, radiation, or other immunosuppressive therapy, were included in the study. All MDS patients, upon entering the study, had >20% blasts in bone marrow that had already transformed to AML. The patients were attended by the hematology departments of four tertiary-care Greek hospitals. Only patients with a bone marrow biopsy at baseline, as part of their routine initial diagnostic workup, were included. For each patient, at least two extra backup slides of bone marrow were saved for iron staining. Exclusion criteria included estimated life expectancy of less than 4 weeks and evidence for active fungal infection at presentation. The study protocol was approved by the Ethics Committee of each participating hospital.

The following data were recorded at baseline: demographics, type of malignancy, neutropenia, significant corticosteroid use, diabetes mellitus, history of blood transfusions, and laboratory assessments obtained as part of routine clinical care, such as white blood cell count, absolute neutrophil count, serum hemoglobin, platelet count, and serum albumin, iron, and ferritin levels. Available chest X-rays or CT scans were reviewed. We defined neutropenia as <500 polymorphonuclear cells/mm^3^ and profound neutropenia as <100 polymorphonuclear cells/mm^3^ for >10 days. Significant corticosteroid use was defined as the use of >600 mg of prednisone equivalent for at least 1 month prior to the diagnosis of IFI or during the follow-up for patients who did not develop IFIs.

The initial bone marrow aspirate smears were stained with Sigma-Aldrich pearl blue iron stain. A standardized scoring system (0–4) was used afterward to evaluate bone marrow iron store (BMIS) [24]. More specifically, scores of 0, 1, and 2 indicated the absence or a small or moderate amount of iron within the bone marrow specimen studied, respectively, whereas scores of 3 and 4 indicated moderate or increased stainable iron stores, respectively. A score of 3 or more was considered an increased BMIS. Almost all patients (95/98) were receiving mold-active antifungal prophylaxis at baseline and before starting chemotherapy.

All patients enrolled in the study were followed prospectively for 12 months for the development of proven or probable invasive fungal infection. Proven and probable IFIs were determined in accordance with the revised European Organization for Research and Treatment of Cancer/Mycoses Study Group (EORTC/MSG) criteria [25]. Briefly, any patient with a fever and neutropenia, and definitive evidence of active fungal infection, such as a positive culture, a positive histopathologic, cytopathologic, or direct microscopic examination of a biopsy specimen, was considered as having proven fungal infection. On the other hand, patients with a neutropenic fever, with no proven IFI but with radiological findings compatible with IFI and/or two consecutive positive galactomannans (for IA), were classified as having a probable fungal infection. As date of IFI onset was considered the date of the first signs or symptoms attributable to IFI or the date of the laboratory diagnosis.

For descriptive statistics, we used mean (SD) for normally distributed continuous variables, median (25–75th percentile) for non-normally distributed continuous variables, and absolute (N) and relative (%) frequencies for categorical variables. The Kaplan–Meier curves for IFI development among patients with BMIS < 3 or ≥ 3 were produced. Modeling risk factors that were independently associated with the development of IFI within one year were found by using univariate and multivariable logistic regression analyses for continuous variables and exact logistic regression for categorical variables. Multivariate logistic regression was also performed to investigate the association of increased baseline BMIS with the development of IFI during the follow-up period, adjusted for known risk factors for IFIs, such as neutropenia (or profound neutropenia alternatively), corticosteroid use, diabetes mellitus, malnutrition, and baseline levels of serum iron and serum ferritin. Although results from univariate analysis did not identify any independent risk factors for IFI development, we proceeded to a multivariate analysis, as it is possible, although uncommon, for an association to become significant only after considering all variables [26]. We applied two sensitivity analyses, excluding the patients with MDS in the first and excluding the patients with invasive candidiasis in the second, and applied the same models. Odds ratios (ORs) and 95% confidence intervals (95% CIs) were reported, and the level of statistical significance was set to 0.05. All analyses were performed with Stata 14 software (Stata Corp., College Station, TX, USA).

## 3. Results

**Figure 1:**
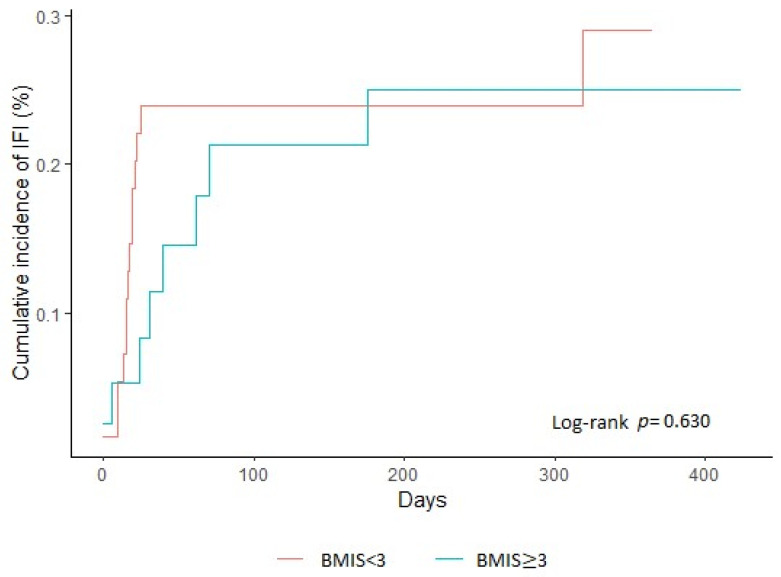
Cumulative incidence of invasive fungal infection diagnosis over 12 months after enrolment.

A total of 129 patients were screened for the study; 31 patients were excluded, because of the non-availability of at least two slides for iron staining, mainly due to a non-productive bone-marrow aspiration (dry tap). The demographics of the 98 patients included in the study are shown in Table 1. A total of 76 (77.6%) patients had AML and 22 (22.4%) had MDS in transformation. The vast majority of patients (95/98) were receiving antifungal prophylaxis. During the one-year follow-up period, 22 (22.4%) patients developed an IFI at a median time of 21 days after baseline assessment (range 0–319 days). The Kaplan–Meier curve for IFI development among patients with a BMIS score < 3 compared to those with a BMIS score ≥ 3 is presented in Figure 1. No statistically significant difference in the cumulative incidence of IFIs between the two groups was observed (log-rank = 0.630). IFIs were mainly invasive aspergillosis (*n* = 16) and characterized mainly as probable (*n* = 12) (Table 2). The group of patients who developed IFI (*n* = 22) was comparable to the group who did not develop IFI (*n* = 76), regarding age, sex, corticosteroid use, diabetes mellitus, and hypoalbuminemia, at baseline (Table 1). Compared to patients who did not develop IFIs, patients with IFIs more frequently had newly diagnosed acute leukemia (95.5% vs. 73.7%), neutropenia (40.9% vs. 23.7%), and profound neutropenia (27.3% vs. 13.2%) at diagnosis. It should be noted that after the initial diagnosis, all patients received induction chemotherapy, and all developed chemotherapy-induced neutropenia. Regarding the markers of iron overload, the two groups had comparable baseline serum levels of iron and ferritin (Table 1). A BMIS score ≥ 3, which is a more reliable marker of iron overload, was observed in similar frequency in the two groups (36.4% among patients with IFIs vs. 40.8% among patients without IFIs). Univariate analysis showed that the only independent predictor for IFI was the diagnosis of acute leukemia at baseline, indicating that patients with acute leukemia had 7.40 (95% CI: 1.05–325.42) times higher odds of developing an IFI compared to those with MDS in transformation. However, the subsequent multivariate analysis failed to reveal any statistically significant predictor of IFIs after adjustment for variables such as neutropenia, corticosteroid use, diabetes mellitus, malnutrition, and baseline levels of serum iron and serum ferritin (Table 1). Both univariate and multivariate analyses showed that an increased BMIS score (≥3) at baseline was not an independent risk factor for IFIs (Table 1). Baseline BMIS score (≥3) was not an independent risk factor after replacing neutropenia with profound neutropenia at diagnosis in the same statistical model (Table S1). As a sensitivity analysis and to have a more homogenous population regarding the risk for IFIs, we applied the same model, including only patients with acute leukemia and excluding patients with MDS in transformation (Table S2). Similarly, this model did not identify the BMIS as an independent risk factor for IFIs in this AML-only patient population. Similar results were provided by the sensitivity analysis when we excluded patients with invasive candidiasis (Table S3).

## 4. Discussion

Researchers have shown previously in laboratory studies, small case series, and retrospective clinical studies that iron overload might be associated with increased risk for IFIs, including invasive candidiasis and invasive mold infections, among patients with hematological malignancies [7,16,18,27,28]. This prospective study was designed to investigate whether iron overload, as measured by the BMIS score, is associated with increased risk for IFIs among patients with AML or MDS in transformation, and multivariate analysis showed no evidence that baseline iron overload is an independent risk factor for IFIs in this specific population of heavily immunocompromised patients. We also performed confirmatory post hoc analyses that included only patients with AML, excluded patients with candidemia, or adjusted only for profound neutropenia at diagnosis. These models similarly failed to show that a BMIS score ≥3 at baseline is an independent risk factor for IFIs.

We prospectively studied 98 patients with acute leukemia or MDS in transformation, and we found that a baseline BMIS score ≥ 3 was not associated with increased risk for IFIs. Kontoyiannis et al. retrospectively evaluated the BMIS score in patients with leukemia as well as recipients of allogeneic HSCT with IA (*n* =33) and those without IA (*n* = 33) and found that most patients with IA (70%) had BMIS scores ≥ 3 compared with the control patients (16%) (*p* < 0.0001). Increased BMIS score was found to be an independent risk factor for IA on multivariate analysis (*p* < 0.0001) [7]. The contradictory findings of the two studies might be explained by the retrospective nature of the latter study as well as from the differences in the composition of the studied populations, as in our study, there are no HSCT recipients, and all our patients were newly diagnosed, whereas Kontoyiannis’s study also included patients refractory to treatment and/or relapsed. Moreover, Kontoyiannis et al. included only patients with IA, whereas, in our study, we examined all IFIs, including invasive candidiasis and invasive mold infections. To produce comparable data, we ran a sensitivity analysis using as an endpoint only the development of IA, but a BMIS ≥ 3 still did not emerge as an independent risk factor (Table S3). In another clinical study, Miceli et al. studied the BMIS in patients with multiple myeloma before autologous HSCT and found that iron overload, as expressed by a high BMIS score, is a risk factor for serious bacterial infections after HSCT [27]. Yet the study did not report any IFIs among the MM patients, who are at low risk for fungal infections.

Our patient population consisted of patients with newly diagnosed AML/MDS, who typically have a relatively low (typically < 10%) risk of IFI compared to refractory/relapsed AML, yet we report relatively high rates of IFIs. A possible explanation is that the particular conditions in Greek hospitals, especially during the recent financial crisis and the subsequent SARS-CoV-2 pandemic favored high rates of aspergillosis, as has been shown in a recent study [29]. On the other hand, our patient population differs from the heavily treated or relapsed patients of similar published studies, where iron overload is nothing more than a marker of poor response of AML to therapy, which is the driver of fungal risk and, therefore, could not be an independent factor [30].

We found that baseline serum levels of iron and ferritin did not differ significantly between the group of patients who subsequently developed an IFI and those who did not. High levels of ferritin are present as an inflammatory marker on many occasions other than infections. Inflammation, in both pediatric and adult patients with AML, has been found to remodel the bone marrow immune microenvironment resulting in worse outcomes, but not propensity to fungal infections [31]. In line with this, Tanaka et al. studied 190 patients with acute leukemia or MDS who have had HSCT and reported that high levels of pre-transplant ferritin did not influence the incidence of infection but were associated with worse survival. Similar data were reported by Styczynski et al. amongst pediatric HSCT recipients [32]. Iglesias-Olma and colleagues reported in a retrospective study of 74 patients with hematological malignancies that patients who developed IFI had increased serum iron and ferritin levels compared to patients without IFI [18]. However, this study was retrospective, and only 30 of the 74 patients had acute leukemia. In a study of 15 patients with allogeneic HSCT, Maertens et al. showed that the number of red cell transfusions and the mean levels of serum ferritin were significantly higher among the five patients with mucormycosis compared with the 10 matched controls [28]. Yet, the small number of patients limits the validity of the findings. Finally, Karp and Merz [22] reported that among 70 patients with acute leukemia, the baseline total iron binding capacity was significantly lower among patients who developed IFIs (*n* = 54) compared to those who did not (*n* = 16). Although this study was prospective, only 13 had documented IFI (12 had candidiasis), while patients with *Candida* esophagitis or candiduria were classified as suspected to have IFI.

In our study, neutropenia at the time of initial diagnosis (i.e., diagnostic bone marrow aspiration/biopsy) and before chemotherapy did not emerge as an independent risk factor for IFIs. However, the patients received induction chemotherapy immediately after diagnosis, and all developed chemotherapy-induced neutropenia by day 14 of the study follow-up. Therefore, all patients are considered neutropenic and of high risk for IFIs, as the association of chemotherapy-induced neutropenia with the development of IFIs is well established [1]. The risk for IFIs due to neutropenia at diagnosis does not necessarily reflect the risk for IFIs due to chemotherapy-induced neutropenia.

Our study has limitations, including the relatively small number of patients and the fact that most IFIs are classified as probable based on the revised EORTC/MSG criteria. Also, only one experienced hematologist assessed the BMIS, which is a semiquantitative grading system, and interobserver variability may exist. Yet the method is highly reproducible and validated for the diagnosis of moderate/severe iron overload [33]. We also did not consider the possible heterogeneity between the participating centers regarding the availability of diagnostic modalities. Finally, the high incidence of IFIs, combined with the relatively low number of patients and the fact that all patients developed chemotherapy-induced neutropenia and all were under antifungal prophylaxis, might have blurred the effect of well-known risk factors for IFIs. Yet, this is, to our knowledge, the first prospective, multi-center study designed to investigate whether iron overload, as measured by the BMIS score, is associated with increased risk for IFIs among high-risk patients with AML or MDS in transformation.

## 5. Conclusions

In conclusion, in our study population of patients with AML or MDS in transformation, baseline iron overload, as expressed by a BMIS score ≥3, was not an independent risk factor for IFIs.

## Figures and Tables

**Table 1:** Characteristics of patients who developed or did not develop invasive mold infection during the follow-up period of one year after the initial bone-marrow aspiration.

Characteristics at Diagnosis of Hematological Malignancy	No IFI (*n* = 76)	Patients with IFI (*n* = 22)	Univariate Analysis		Multivariate Analysis	
			OR (95% CI)	*p*	OR (95% CI)	*p*
Age (median, 25–75th percentile, years)	67.5 (53.5–75.0)	58 (47–65)	-	-	-	-
Male sex	44/76 (57.9%)	14/22 (63.6%)	-	-	-	-
AML	56/76 (73.7%)	21/22 (95.5%)	7.40 (1.05–325.42)	0.037	7.96 (0.94–67.34)	0.057
Neutropenia at diagnosis (<500 cells/mm^3^)	18/76 (23.7%)	9/22 (40.9%)	2.21 (0.71–6.72)	0.174	1.97 (0.68–5.74)	0.214
Profound neutropenia at diagnosis (<100 cells/mm^3^)	10/76 (13.2%)	6/22 (27.3%)	2.45 (0.63–8.83)	0.186	-	-
Corticosteroid use	17/76 (22.4%)	5/22 (22.7%)	1.02 (0.26–3.47)	>0.999	1.11 (0.32–3.82)	0.874
Diabetes mellitus	7/76 (9.2%)	2/22 (9.1%)	0.99 (0.09–5.76)	>0.999	1.01 (0.17–6.06)	0.995
Malnutrition (albumin < 3 mg/dL)	25/76 (32.9%)	7/22 (31.8%)	0.95 (0.29–2.88)	>0.999	1.13 (0.37–3.46)	0.828
BMIS ≥ 3	31/76 (40.8%)	8/22 (36.4%)	0.83 (0.27–2.43)	0.807	0.88 (0.31–2.50)	0.805
Baseline serum iron levels (median, 25–75th percentile, mg/dL)	125.5 (79.5–170.5)	114.0 (92.0–156.0)	1.00 (0.98–1.00)	0.334	1.00 (0.99–1.00)	0.346
Baseline serum ferritin levels (median, 25–75th percentile, mg/dL)	678.2 (233.4–1012.0) (*n* = 75)	873.3 (386.1–1225.6)	1.00 (0.99–1.00)	0.911	1.00 (0.99–1.00)	0.519

IFI: invasive fungal infection; AML: acute myeloid leukemia; and BMIS: bone marrow iron store.

**Table 2:** Number of cases of invasive fungal infections developed during the 12-month follow-up period in the study population.

Invasive Fungal Infection	Proven *	Probable *	Total
Invasive candidiasis/candidemia	5	0	5
Invasive aspergillosis	4	12	16
Mucormycosis	1	0	1
Total	10	12	22

* According to the revised EORTC/MSG definitions [25].

## Data Availability

The raw data supporting the conclusions of this article will be made available by the authors, without undue reservation to any qualified researcher.

## Supplementary Materials

The following supporting information can be downloaded at: https://www.mdpi.com/article/10.3390/jof9070748/s1, Table S1: Results from multivariate analysis for the investigation of the association of increased BMIS and development of invasive fungal infection, adjusted for profound neutropenia, corticosteroid use, diabetes mellitus, malnutrition and baseline levels of serum iron and serum ferritin; Table S2: Characteristics of patients with acute myeloid leukemia who developed or did not develop invasive fungal infection during the follow up period of one year after the initial bone-marrow aspiration and results from univariate and multivariate models. Patients with myelodysplastic syndrome have been excluded; Table S3: Characteristics of patients who developed or not invasive aspergillosis, during the follow up period of one year after the initial bone-marrow aspiration and results from univariate and multivariate models. Patients with invasive candidiasis have been excluded.

## Abbreviations

IA: invasive aspergillosis; MDS: myelodysplastic syndrome; AML: acute myeloid leukemia; IFI: invasive fungal disease; BMIS: bone marrow iron store.
