# Supplementary material for: Bone Marrow Iron Stores Are Not Associated with Increased Risk for Invasive Fungal Infections in Patients with Newly Diagnosed Acute Leukemia or Myelodysplastic Syndrome in Transformation: Is There a Relationship?
Source: J Fungi (Basel). 2023 Jul 14;9(7):748. doi: 10.3390/jof9070748 (PMC10381291; doi:10.3390/jof9070748)
Supplement: Supplementary file 1 [file jof-09-00748-s001.zip › jof-2466781-supplementary.pdf]

Supplement

Table S1. Results from multivariate analysis for the investigation of the association of increased BMIS and development of invasive fungal infection, adjusted for profound neutropenia, corticosteroid use, diabetes mellitus, malnutrition and baseline levels of serum iron and serum ferritin

**Risk Factor**	**Multivariate analysis**	
	**OR (95% CI)**	***P***
AML	7.95 (0.93- 67.83)	0.058
Profound neutropenia (<100 cells / mm^3)	2.17 (0.62- 7.61)	0.228
Corticosteroid use	1.04 (0.3- 3.65)	0.950
Diabetes mellitus	0.94 (0.16- 5.58)	0.943
Malnutrition (albumin < 3 mg/dL)	1.09 (0.35- 3.41)	0.876
BMIS $\geq$ 3	0.85 (0.29- 2.43)	0.755
Baseline serum iron levels (median, 25th-75th percentile, mg/dL)	1.00 (0.99- 1.00)	0.354
Baseline serum ferritin levels (median, 25th-75th percentile, mg/dL)	1.00 (0.99- 1.00)	0.515

BMIS: bone marrow iron stores score, AML: acute myeloid leukemia, OR: odds ratio, CI: confidence intervals

Table S2. Characteristics of patients with acute myeloid leukemia who developed or did not develop invasive fungal infection during the follow up period of one year after the initial bone-marrow aspiration and results from univariate and multivariate models. Patients with myelodysplastic syndrome have been excluded.

Characteristics at diagnosis of hematological malignancy	No IFI (n=56)	IFI (n=21)	Univariate analysis		Multivariate analysis			
			OR (95% CI)	*P*	OR (95% CI) *	*P**	OR (95% CI) †	*P*†
Age (median, 25th-75th percentile, years)	66.0 (53.0-74.0)	58.0 (47.0-65.0)	-	-	-	-	-	-
Male sex	34/56 (60.7%)	13/21 (61.9%)	-	-	-	-	-	-
Neutropenia (<500 cells / mm^3)	14/56 (25.0%)	9/21 (42.9%)	2.22 (0.68-7.27)	0.216	2.20 (0.72-6.68)	0.164	-	-
Profound neutropenia (<100 cells / mm^3)	8/56 (14.3%)	6/21 (28.6%)	2.37 (0.58-9.31)	0.267	-	-	2.34 (0.64-8.57)	0.199
Corticosteroid use	11/56 (19.6%)	5/21 (23.8%)	1.27 (0.30-4.79)	0.909	1.26 (0.34-4.64)	0.732	1.19 (0.32-4.45)	0.791
Diabetes mellitus	5/56 (8.9%)	2/21 (9.5%)	1.07 (0.09-7.26)	>0.999	0.98 (0.15-6.47)	0.981	0.90 (0.14-5.85)	0.908
Malnutrition (albumin < 3 mg/dL)	15/56 (26.8%)	7/21 (33.3%)	1.36 (0.39-4.50)	0.764	1.38 (0.43-4.41)	0.585	1.34 (0.42-4.35)	0.621
BMIS ≥ 3	22/56 (39.3%)	7/21 (33.3%)	0.78 (0.23-2.46)	0.837	0.73 (0.24-2.23)	0.586	0.70 (0.23-2.15)	0.535
Baseline serum iron levels	121.5 (79.5-170.5)	113.0 (92.0-154.0)	0.99 (0.98-1.00)	0.233	0.99 (0.98-1.00)	0.195	0.99 (0.98-1.00)	0.198

**Characteristics at diagnosis of hematological malignancy**	**No IFI (n=56)**	**IFI (n=21)**	**Univariate analysis**		**Multivariate analysis**			
(median, 25th-75th percentile, mg/dL)								
Baseline serum ferritin levels (median, 25th-75th percentile, mg/dL)	524.4 (225.1-846.9)	861.6 (386.1-1202)	1.00 (0.99-1.00)	0.690	1.00 (0.99-1.00)	0.574	1.00 (0.99-1.00)	0.559
*Model adjusted for neutropenia; †model adjusted for profound neutropenia								

IFI: invasive fungal infection, BMIS: bone marrow iron stores score, OR: odds ratio, CI: confidence intervals

Table S3. Characteristics of patients who developed or not invasive aspergillosis, during the follow up period of one year after the initial bone-marrow aspiration and results from univariate and multivariate models. Patients with invasive candidiasis have been excluded.

Characteristics at diagnosis of hematological malignancy	No IA (n=76)	IA (n=17)	Univariate analysis		Multivariate analysis			
			OR (95% CI)	*P*	OR (95% CI) *	*P**	OR (95% CI) †	*P*†
Age (median, 25th-75th percentile, years)	67.5 (53.5-75)	55 (47-65)	-	-	-	-	-	-
Male sex	44/76 (57.9%)	11/17 (64.7%)	-	-	-	-	-	-
AML	56/76 (73.7%)	16/17 (94.1%)	5.64 (0.77-251.42)	0.107	5.73 (0.67-49.08)	0.111	5.89 (0.69-50.5)	0.106
Neutropenia (<500 cells / mm^3)	18/76 (23.7%)	7/17 (41.2%)	2.23 (0.63-7.66)	0.224	1.96 (0.6-6.43)	0.267	-	-
Profound neutropenia (<100 cells / mm^3)	10/76 (13.2%)	4/17 (23.5%)	2.01 (0.40-8.46)	0.278	-	-	1.73 (0.41-7.34)	0.459
Corticosteroid use	17/76 (22.4%)	3/17 (17.7%)	0.75 (0.12-3.14)	>0.999	0.82 (0.19-3.45)	0.785	0.79 (0.19-3.38)	0.753
Diabetes mellitus	7/76 (9.2%)	1/17 (5.9%)	0.62 (0.01-5.40)	>0.999	0.76 (0.08-7.37)	0.815	0.68 (0.07-6.37)	0.732
Malnutrition (albumin < 3 mg/dL)	25/76 (32.9%)	5/17 (29.4%)	0.85 (0.21-2.96)	>0.999	0.96 (0.27-3.39)	0.948	0.98 (0.27-3.53)	0.970
BMIS ≥ 3	31/76 (40.8%)	8/17 (47.1%)	1.29 (0.39-4.23)	0.787	1.35 (0.44-4.13)	0.602	1.32 (0.43-4.06)	0.626

**Characteristics at diagnosis of hematological malignancy**	**No IA (n=76)**	**IA (n=17)**	**Univariate analysis**		**Multivariate analysis**			
Baseline serum iron levels (median, 25th-75th percentile, mg/dL)	125.5 (79.5-170.5)	1128 (95-156)	1.00 (0.99-1.01)	0.584	1 (0.99-1.01)	0.570	1 (0.99-1.01)	0.585
Baseline serum ferritin levels (median, 25th-75th percentile, mg/dL)	678.2 (233.4-1012.0)	688.1 (262.3-1102.5)	1.00 (0.99-1.00)	0.974	1.00 (0.99-1.00)	0.738	1.00 (0.99-1.00)	0.710
*Model adjusted for neutropenia; †model adjusted for profound neutropenia								

IA: invasive aspergillosis, BMIS: bone marrow iron stores score, OR: odds ratio, CI: confidence intervals
